# Sedentary behaviours and its association with bone mass in adolescents: the HELENA cross-sectional study

**DOI:** 10.1186/1471-2458-12-971

**Published:** 2012-11-13

**Authors:** Luis Gracia-Marco, Juan P Rey-López, Alba M Santaliestra-Pasías, David Jiménez-Pavón, Ligia E Díaz, Luis A Moreno, German Vicente-Rodríguez

**Affiliations:** 1 School of Sport and Health Sciences, University of Exeter, Heavitree Road, Exeter, EX1 2LU, UK; 2 GENUD (Growth, Exercise, NUtrition and Development) Research Group, University of Zaragoza, Zaragoza, Spain; 3 Children Institute, School of Medicine, University of São Paulo, São Paulo, Brazil; 4 Facultad de Ciencias de la Salud, Universidad de Zaragoza, C/Domingo Miral s/n, 50009, Zaragoza, Spain; 5 Department of Physiology, School of Medicine, University of Granada, Granada, Spain; 6 Immunonutrition Research Group, Department of Metabolism and Nutrition, Institute of Food Science, Technology and Nutrition (ICTAN), Spanish National Research Council (CSIC), Madrid, Spain; 7 Faculty of Health and Sport Science (FCSD), Department of Physiatry and Nursing, University of Zaragoza, Ronda Misericordia 5, 22001, Huesca, Spain

**Keywords:** Bone health, Sedentary behaviours, Adolescents, Physical activity and extra-curricular participation in sports

## Abstract

**Background:**

We aimed to examine whether time spent on different sedentary behaviours is associated with bone mineral content (BMC) in adolescents, after controlling for relevant confounders such as lean mass and objectively measured physical activity (PA), and if so, whether extra-curricular participation in osteogenic sports could have a role in this association.

**Methods:**

Participants were 359 Spanish adolescents (12.5-17.5 yr, 178 boys,) from the HELENA-CSS (2006–07). Relationships of sedentary behaviours with bone variables were analysed by linear regression. The prevalence of low BMC (at least 1SD below the mean) and time spent on sedentary behaviours according to extracurricular sport participation was analysed by Chi-square tests.

**Results:**

In boys, the use of internet for non-study was negatively associated with whole body BMC after adjustment for lean mass and moderate to vigorous PA (MVPA). In girls, the time spent studying was negatively associated with femoral neck BMC. Additional adjustment for lean mass slightly reduced the negative association between time spent studying and femoral neck BMC. The additional adjustment for MVPA did not change the results at this site. The percentage of girls having low femoral neck BMC was significantly smaller in those participating in osteogenic sports (≥ 3 h/week) than in the rest, independently of the cut-off selected for the time spent studying.

**Conclusions:**

The use of internet for non-study (in boys) and the time spent studying (in girls) are negatively associated with whole body and femoral neck BMC, respectively. In addition, at least 3 h/week of extra-curricular osteogenic sports may help to counteract the negative association of time spent studying on bone health in girls.

## Background

It is well known that acquiring a high bone mass during childhood and adolescence is a key determinant for adult skeletal health [1]. Although genetics greatly determine bone mass [2], environmental and lifestyle factors, such as physical activity (PA) have important osteogenic effects [3]. There is strong evidence that exercise has important osteogenic effects, mainly when high-impact and weight-bearing PA occur [4]. Increased bone mass during childhood and adolescence has been associated to the frequency, intensity and type of PA [3,5-8]. This phenomenon is related to the mechanostat theory [9,10], suggesting that both exercise and PA could drive to a direct osteogenic effect on bone mass and an indirect osteogenic effect by increasing muscle size and strength and hence the tensions generated on bones [4].

A common misconception is to identify as sedentary those subjects that do not comply with PA guidelines. Guidelines for children and adolescents, recommend that young people should accumulate at least 60 minutes (up to several hours) of moderate to vigorous PA (MVPA) per day [11]. However, the term sedentary should only be used when a subject stay several hours in positions requiring very low energy expenditure (<1.5 metabolic equivalent, MET) [12].

To the best of our knowledge, there is a lack of information regarding the association between sedentary behaviours and bone health. A recent study showed that sedentary behaviours may have a role on bone health, and also that the deleterious health outcomes caused by sedentary behaviours differ from those that are associated with inadequate PA levels [13]. Vicente-Rodríguez et al. recently showed that adolescents from the AVENA study who watched television (TV) more than 3 hours/day, had an increased risk of low whole body bone mineral content (BMC) [14]. Nonetheless, the association was only observed in males and disappeared when participation in extra-curricular sports was taken into account, suggesting that the negative consequences of sedentary behaviours on bone health can be counteracted by extra-curricular sport participation. In the present study, we do so but taking into account a large list of sedentary behaviours and bone mass related-variables and using objectively measured PA and lean mass as confounders in our analyses. A better understanding of the sedentary behaviour-bone health association is of great importance, especially on a key period as adolescence and, in regions with clinical relevance in the diagnosis of osteoporosis, such as femoral neck and lumbar spine [1].

The Healthy Lifestyle in Europe by Nutrition in Adolescence Cross-Sectional Study (HELENA-CSS) retrieved interesting information on sedentary behaviours and bone health, and provides a good opportunity to explore the association between these variables in adolescence, after controlling for key confounders, such as lean mass [15] and objectively measured PA [3].

In this study, we aimed to examine whether time spent on different sedentary behaviours (i.e. TV viewing, computer games, console games, internet for non-study, internet for study, study and total sedentary time) is associated with BMC (i.e. whole body, lumbar spine and femoral neck) in adolescents, after controlling for relevant confounders such as lean mass and objectively measured PA, and if so, whether extra-curricular participation in osteogenic sports could have a role in this association.

## Methods

### Participants

The HELENA-CSS used harmonized and well standardized methods of measurement in European adolescents in 2006–07 [16], which have been described in detail elsewhere [17]. Briefly, the HELENA-CSS aimed to describe the lifestyle and nutritional status among European adolescents [16] and took place between October 2006 and December 2007 in ten European cities. To guarantee that heterogeneity of social background of the population, schools were randomly selected after stratification on school zone or district. Up to three classes from two grades were selected per school. In this report, we focus on the sample from Zaragoza (Spain), one of the 10 centres (cities) involved in the HELENA-CSS, where bone mass was measured by dual energy X-ray absorptiometry (DXA). Inclusion criteria were to have valid data on height, sexual maturation, DXA (bone and lean mass), objectively measured PA and self-report of sedentary behaviours. Twenty-four adolescents from Zaragoza were excluded due to some missing data in these variables. A total of 178 boys and 181 girls were finally included in the analyses. The participants included (n=359) in our analyses did not differ from those excluded from Zaragoza (n=24) in weight, height, body mass index (BMI) and time spent in any sedentary behaviour. The study was performed following the ethical guidelines of the Declaration of Helsinki 1975 (as revised in 1983). The study was approved by the Ethics Committee of Clinical Research from the Government of Aragón (CEICA, Spain) [18]. Written informed consent was obtained from both, adolescents and their parents.

### Sedentary behaviours

A self-report sedentary behaviour questionnaire was administered during the school hours. Adolescents reported time watching TV, playing with computer games, playing with console games, surfing by internet for reasons other than study, surfing by internet due to study reasons, and studying (non-school time) for week and weekends days, selecting one of the following categories: 1) 0 min, 2) >0–30 min, 3) >30–60 min, 4) >60–120 min, 5) >120–180 min, 6) >180–240 min and 7) >240 min. We estimated the sedentary minutes per day as follows: category 1 = 0 min, 2 = 15 min, 3 = 45 min, 4 = 90 min, 5 = 150 min, 6 = 210 min and 7 = 241 min, respectively. These cut-offs have been previously used [19]. Total sedentary time was computed as the sum of the time spent on the reported sedentary behaviours. The HELENA sedentary behaviour questionnaire is a reliable questionnaire to be used in adolescents [19].

### Body composition measurements

International guidelines for anthropometry in adolescents were used in the HELENA-CSS [20]. While the participants were barefoot and clad in light indoor clothing, body weight (Kg) and height (cm) were measured with an electronic scale (Type SECA 861), precision 100 g, range 0–150 Kg, and a stadiometer (Type Seca 225), precision 0.1 cm, range 70–200 cm, respectively.

Osseous and soft tissues of the adolescents were measured with DXA, using a paediatric version of the software QDR-Explorer (Hologic Corp., Software version 12.4, Bedford, MA, USA). A lumbar spine phantom was used for assuring quality control of measurement as recommended by the manufacturer. For the whole body measurement, adolescents were scanned in supine position and the scans were performed at high resolution [8]. The bone mineral density (BMD; g·cm^–2^), area (cm^2^), fat mass (g) and lean mass (g) [body mass – (fat mass + bone mass)] were determined for each individual from total and regional analysis of the whole body scan. Bone mineral content (g) was calculated using the formula BMC = BMD·area. Two additional examinations were conducted to estimate bone mass at the lumbar spine (mean L1-L4) and hip sub-regions (trochanter, intertrochanter and femoral neck) as previously described [21].

The coefficients of variation (CV) of the DXA in our lab were calculated for regional analysis of the complete body scan in 49 adolescents with repositioning. The CV were: 2.3% for BMC; 1.3% for BMD; 2.6% for bone area and 1.9% for lean mass [15].

### Pubertal status assessment

A physician examined the adolescents in order to classify them in one of the five stages proposed by Tanner and Whitehouse [22].

### Physical activity

Uniaxial accelerometers (Actigraph GT1M, Manufacturing Technology Inc. Pensacola, FL, USA) were used to assess PA as described previously [23]. In this study, the interval of time (epoch) was set at 15 seconds. The time spent (minutes/day) at moderate PA (MPA) [3–6 METs] was calculated based upon a cut-off of 2000–3999 counts per minute (cpm) [24], which is equivalent to the intensity of a brisk walk (4.5 km^.^h^-1^). The time spent (minutes/day) at vigorous PA (>6 METs) was calculated based upon a cut-off of 4000 cpm. Further, MVPA (>3 METs) was calculated as the sum of moderate and vigorous PA. The cut-offs to define the intensity categories are similar to those used in previous studies [25].

Participants were classified as non-active adolescents (<60 minutes/day of MVPA) and active adolescents (≥60 minutes/day of MVPA) according to the recent guidelines launched by the U.S. Department of Health and Human Services and other medical institutions [11].

### Extra-curricular participation in sports

Adolescents were asked about their extra-curricular participation in sports and the average time per week. Once the answers were obtained, adolescents were classified in two different groups: 1) active adolescents: those doing ≥3 h/week of osteogenic sports (i.e. football, basketball, tennis, etc.); and 2) rest of adolescents: those doing <3 h/week of osteogenic sports, or doing non-osteogenic sports (i.e. swimming, cycling, skating, etc.), or not doing any extra-curricular sport.

The selection of this cut-off is based on scientific evidence. Children (9–12 yr) doing ≥3 h/week of extra-curricular sport increased total lean and bone mass to a greater extent than those children who did not participate in extra-curricular sports [5,7].

### Statistics

After square root transformation of the continuous variables of TV viewing, use of computer games and time spent studying and natural logarithm transformation of console games, internet for non-study, internet for study and the total sedentary time, all variables showed a normal distribution (established by using Kolmogorov-Smirnov tests). Descriptive data were assessed by one-way analysis of variance (ANOVA) for normally distributed variables and by U Mann Whitney for non-normally distributed variables. Relationships of sedentary behaviours (i.e. the continuous variables of TV viewing, use of computer games, console games, internet for non-study, internet for study, time spent studying and total sedentary time) with different bone mass related variables (i.e. whole body, lumbar spine and femoral neck) were analysed using linear regression models, including height and sexual maturation as covariates (model 1). Model 2 included model 1 + total lean mass to test the role of lean mass in this association. Model 3 included model 2 + MVPA to test the role of MVPA in this association. All residuals showed a satisfactory pattern.

Additionally, BMC and BMD z-scores were calculated using a reference standard obtained by age and gender [26] in regions with clinical relevance in the diagnosis of osteoporosis and with significant associations in regression analyses (i.e. femoral neck). Once obtained, adolescents with low BMC [at least 1 standard deviation (SD) below the mean] were selected to assess the prevalence of low BMC according to time of study in active girls vs. the rest of girls (Chi-square tests). As there is no consensus in the literature about the appropriate cut-offs for sedentary behaviours (i.e. time of study), two different cut-offs were used: (low study; <2 h/day and high study; ≥ 2 h/day) and (low study; <3 h/day and high study; ≥ 3 h/day).

All the analyses were performed using the Statistical Package for Social Sciences software (SPSS, v. 15.0 for WINDOWS; SPSS Inc., Chicago, IL, USA), and values of p<0.05 were considered statistically significant.

## Results

Table 1 shows descriptive characteristics by gender. Results showed that there were no differences between boys and girls in age, lumbar spine BMC, time spent on TV viewing and total sedentary time; however, most traits differed by gender.

**Table 1:** Descriptive characteristics of the studied adolescents (n=359)

**Variables**	**All (n=359)**	**Boys (n=178)**	**Girls (n=181)**
Age (y)	14.8 ± 1.2	14.8 ± 1.2	14.8 ± 1.1
Sexual maturation (I/II/III/IV/V) (%)	(0/2/6/13/79)	(0/3/9/20/68)	(0/1/4/6/89)*
Body mass (Kg)	58.3 ± 13.1	62.2 ± 15.3	54.6 ± 9.1*
Height (cm)	164.6 ± 10.9	168.5 ± 12.2	160.4 ± 7.2*
Whole body lean mass (Kg)	40.5 ± 8.5	45.6 ± 8.5	35.5 ± 4.7*
Whole body fat mass (Kg)	14.8 ± 6.1	13.3 ± 6.5	16.4 ± 5.3*
Whole body BMC ^a^ (g)	1999.34 ± 421.73	2127.21 ± 473.35	1873.61 ± 318.34*
Lumbar spine BMC ^a^ (g)	51.15 ± 12.84	52.13 ± 14.87	50.16 ± 10.38
Femoral neck BMC ^a^ (g)	4.24 ± 0.89	4.63 ± 0.97	3.85 ± 0.61*
MVPA ^b^ (minutes/day)	58.8 ± 24.1	65.7 ± 24.8	49.9 ± 20.4*
TV viewing (minutes/day)	107.1 (62.1-150)	107.1 (57.8-139.3)	107.1 (75–167.1)
Use of computer games (minutes/day)	23.6 (0–60.)	36.4 (12.8-77.1)	7.5 (0–36.4)*
Use of console games (minutes/day)	4.3 (0–36.4)	25.7 (4.3-57.8)	0 (0–4.3)*
Use of internet for non-study (minutes/day)	45 (15–92.1)	36.4 (10.7-75)	57.8 (23.6-107.1)*
Use of internet for study (minutes/day)	15 (9.1-36.4)	15 (0–32.1)	15 (10-7-45)*
Time of study (minutes/day)	90 (36.4-132.9)	75 (23.6-120)	90 (45–150)*
Total sedentary time (minutes/day)	331.1 (242.1-460.2)	327.9 (241.1-457.5)	340.7 (242.1-473.6)

ANOVA was performed for normally distributed variables (mean ± SD) and *U* Mann Whitney for non-normally distributed variables (median and interquartil intervals).

^a^ Bone Mineral Content; ^b^ Moderate to Vigorous Physical Activity.

* Sex differences (p<0.05).

In boys, total sedentary time was negatively associated with whole body BMC (partial corr, -0.157, p=0.043) (Table 2). However, significant differences disappeared after additional adjustment for lean mass (model 2) and MVPA (model 3). In addition, the use of internet for non-study was negatively associated with whole body BMC after additional adjustment for lean mass (model 2) (partial corr, -0.167, p=0.047) and MVPA (model 3) (partial corr, -0.168, p=0.047) (Table 2). No significant associations were found between sedentary behaviours and both lumbar spine (Table 3) and femoral neck (Table 4).

**Table 2:** Linear regression analysis for whole body bone mineral content as regards to sedentary behaviours

**Boys (n=178)**				**Girls (n=181)**		
***Model 1: Adjusted by height and sexual maturation***						
**Sedentary behaviours**^**†**^	**β**^**a**^	**Partial Corr**^**b**^	**P**	**β**^**a**^	**Partial Corr**^**b**^	**P**
TV viewing ^c^	−0.043	−0.057	0.452	0.030	0.040	0.607
Use of computer games ^c^	0.066	0.089	0.242	−0.039	−0.052	0.502
Use of console games ^d^	0.001	0.001	0.988	−0.135	−0.202	0.188
Use of internet for non-study ^d^	−0.067	−0.093	0.269	0.066	0.089	0.280
Use of internet for study ^d^	−0.023	−0.029	0.745	0.097	0.132	0.119
Time of study ^c^	−0.080	−0.08	0.159	−0.129	−0.174	**0.023**
Total sedentary time ^d^	−0.092	−0.157	**0.043**	−0.087	−0.117	0.139
*Model 2: Model 1 + lean mass*						
TV viewing ^c^	0.010	0.022	0.772	0.003	0.005	0.947
Use of computer games ^c^	−0.008	−0.017	0.822	−0.043	−0.072	0.356
Use of console games ^d^	0.046	0.104	0.238	−0.053	−0.101	0.518
Use of internet for non-study ^d^	−0.074	−0.167	**0.047**	0.058	0.097	0.241
Use of internet for study ^d^	−0.029	−0.063	0.484	0.089	0.149	0.055
Time of study ^c^	0.016	0.033	0.663	−0.045	−0.074	0.338
Total sedentary time ^d^	−0.033	−0.074	0.343	−0.021	−0.035	0.662
*Model 3: Model 2 + MVPA*^*e*^						
TV viewing ^c^	0.006	0.012	0.872	0.001	0.002	0.983
Use of computer games ^c^	−0.010	−0.022	0.776	−0.044	−0.073	0.347
Use of console games ^d^	0.043	0.099	0.265	−0.036	−0.067	0.673
Use of internet for non-study ^d^	−0.075	−0.168	**0.047**	0.064	0.109	0.190
Use of internet for study ^d^	−0.034	−0.073	0.421	0.085	0.140	0.071
Time of study ^c^	0.015	0.033	0.673	−0.050	−0.081	0.296
Total sedentary time ^d^	−0.037	−0.083	0.292	−0.024	−0.040	0.613

Significant results are in **bold**.

^**†**^ All sedentary behaviours represent the average of the whole week (min/day).

^a^*β* is the estimated standardized regression coefficient.

^b^ Partial correlation.

^c^ Square-root transformed data.

^d^ Log-transformed data.

^e^ Moderate to vigorous physical activity.

**Table 3:** Linear regression analysis for lumbar spine bone mineral content as regards to sedentary behaviours

**Boys (n=178)**				**Girls (n=181)**		
***Model 1: Adjusted by height and sexual maturation***						
**Sedentary behaviours**^**†**^	**β**^**a**^	**Partial Corr**^**b**^	**p**	**β**^**a**^	**Partial Corr**^**b**^	**P**
TV viewing ^c^	−0.049	−0.066	0.381	0.064	0.082	0.289
Use of computer games ^c^	0.068	0.093	0.224	−0.081	−0.103	0.188
Use of console games ^d^	−0.008	−0.013	0.884	−0.071	−0.085	0.593
Use of internet for non-study ^d^	−0.022	−0.030	0.720	−0.005	−0.006	0.940
Use of internet for study ^d^	−0.054	−0.072	0.424	0.069	0.088	0.303
Time of study ^c^	−0.061	−0.084	0.274	−0.109	−0.139	0.072
Total sedentary time ^d^	−0.073	−0.119	0.127	−0.073	−0.094	0.238
*Model 2: Model 1 + lean mass*						
TV viewing ^c^	−0.010	−0.017	0.828	0.048	0.065	0.400
Use of computer games ^c^	0.013	0.022	0.777	−0.083	−0.113	0.150
Use of console games ^d^	0.024	0.044	0.616	−0.017	−0.021	0.897
Use of internet for non-study ^d^	0.011	0.018	0.813	−0.034	−0.046	0.557
Use of internet for study ^d^	−0.059	−0.101	0.259	0.098	0.132	0.123
Time of study ^c^	0.010	0.016	0.833	−0.056	−0.074	0.342
Total sedentary time ^d^	−0.037	−0.064	0.414	−0.032	−0.044	0.583
*Model 3: Model 2 + MVPA*^*e*^						
TV viewing ^c^	−0.016	−0.026	0.734	0.045	0.061	0.429
Use of computer games ^c^	0.010	0.018	0.819	−0.085	−0.115	0.144
Use of console games ^d^	0.022	0.041	0.643	0.069	0.085	0.602
Use of internet for non-study ^d^	−0.027	−0.049	0.569	−0.003	−0.005	0.957
Use of internet for study ^d^	−0.067	−0.114	0.207	0.095	0.127	0.139
Time of study ^c^	0.009	0.015	0.843	−0.062	−0.083	0.286
Total sedentary time ^d^	−0.041	−0.071	0.364	−0.036	−0.049	0.537

Significant results are in **bold**.

^**†**^ All sedentary behaviours represent the average of the whole week (min/day).

^a^*β* is the estimated standardized regression coefficient.

^b^ Partial correlation.

^c^ Square-root transformed data.

^d^ Log-transformed data.

^e^ Moderate to vigorous physical activity.

**Table 4:** Linear regression analysis for femoral neck bone mineral content as regards to sedentary behaviours

**Boys (n=178)**				**Girls (n=181)**		
***Model 1: Adjusted by height and sexual maturation***						
**Sedentary behaviours**^**†**^	**β**^**a**^	**Partial Corr**^**b**^	**p**	**β**^**a**^	**Partial Corr**^**b**^	**P**
TV viewing ^c^	−0.066	−0.077	0.301	−0.039	−0.045	0.561
Use of computer games ^c^	0.037	0.043	0.577	−0.086	−0.099	0.203
Use of console games ^d^	0.041	0.052	0.553	−0.130	−0.156	0.313
Use of internet for non-study ^d^	−0.071	−0.084	0.320	−0.016	−0.019	0.822
Use of internet for study ^d^	−0.073	−0.082	0.361	−0.053	−0.062	0.466
Time of study ^c^	−0.085	−0.099	0.194	−0.190	−0.221	**0.004**
Total sedentary time ^d^	−0.097	−0.126	0.107	−0.172	−0.201	**0.011**
*Model 2: Model 1 + lean mass*						
TV viewing ^c^	−0.018	−0.026	0.738	−0.063	−0.081	0.294
Use of computer games ^c^	−0.025	−0.035	0.645	−0.090	−0.117	0.133
Use of console games ^d^	0.084	0.120	0.176	−0.069	−0.088	0.574
Use of internet for non-study ^d^	−0.076	−0.110	0.196	−0.025	−0.033	0.691
Use of internet for study ^d^	−0.077	−0.110	0.224	−0.022	−0.028	0.738
Time of study ^c^	0.000	0.000	0.997	−0.119	−0.152	**0.049**
Total sedentary time ^d^	−0.043	−0.061	0.435	−0.113	−0.150	0.058
*Model 3: Model 2 + MVPA*^*e*^						
TV viewing ^c^	−0.021	−0.030	0.695	−0.063	−0.081	0.296
Use of computer games ^c^	−0.026	−0.038	0.577	−0.090	−0.117	0.134
Use of console games ^d^	0.083	0.118	0.183	−0.042	−0.051	0.749
Use of internet for non-study ^d^	−0.076	−0.110	0.197	−0.026	−0.034	0.683
Use of internet for study ^d^	−0.077	−0.108	0.230	−0.024	−0.032	0.712
Time of study ^c^	0.000	0.000	0.999	−0.119	−0.152	**0.049**
Total sedentary time ^d^	−0.045	−0.064	0.413	−0.115	−0.152	0.056

Significant results are in **bold**.

^**†**^ All sedentary behaviours represent the average of the whole week (min/day).

^a^*β* is the estimated standardized regression coefficient.

^b^ Partial correlation.

^c^ Square-root transformed data.

^d^ Log-transformed data.

^e^ Moderate to vigorous physical activity.

In girls, the time spent studying was negatively associated with whole body BMC (partial corr, -0.174, p=0.023) (Table 2). However, significant differences disappeared after additional adjustment for lean mass (model 2) and MVPA (model 3). In addition, there was a negative association between this sedentary behaviour and femoral neck BMC (partial corr, -0.221; p=0.004) (Table 4). Additional adjustment for lean mass (model 2) slightly reduced the negative association between time spent studying and femoral neck BMC (partial corr, -0.152; p=0.049). The additional adjustment for MVPA did not change the results at this site (partial corr, -0.152; p=0.049). No significant associations were found between sedentary behaviours and lumbar spine (Table 3).

Finally and also in girls, the total sedentary time was negatively associated with femoral neck BMC (partial corr, -0.201, p=0.011) (Table 4). Despite the disappearance of significant differences after additional adjustment for lean mass (model 2) (partial corr, -0.150; p=0.058) and MVPA (model 3) (partial corr, -0.152; p=0.056), there was a trend toward significance in both models.

**Figure 1:**
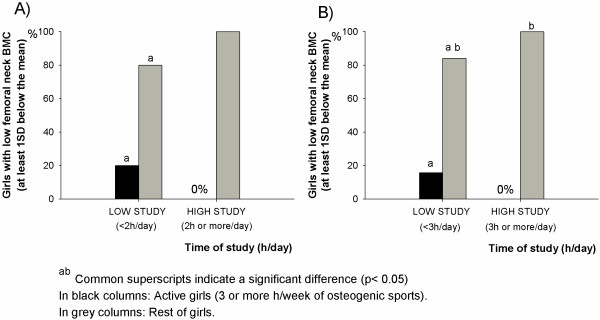
Prevalence of low femoral neck BMC and time of study in active girls (n=30) vs. the rest of girls (n=151).

Due to the importance of femoral neck in the diagnosis of osteoporosis and to the fact that regression analyses showed high and significant associations between this site and time spent studying in girls, complementary analyses were performed. Both in low (<2 h/day) and high (≥2 h/day) time of study, the percentage of girls having low femoral neck BMC (at least 1SD below the mean) was significantly smaller in active girls than in the rest (Figure 1A). The results showed a similar pattern when using the cut-off in 3 h (Figure 1B). Similar results were obtained for whole body and lumbar spine BMC using both cut-offs (data not shown).

## Discussion

The main findings of the present study indicate that (1) some sedentary behaviours, such as the use of internet for non-study in boys and the time spent studying in girls are negatively associated with whole body and femoral neck BMC, respectively, and (2) active girls (≥3 h/week of osteogenic sports) present a lower prevalence for having low femoral neck BMC (at least 1SD below the mean), suggesting that the negative effect on bone mass of a sedentary behaviour such as time for study, could be counteracted by the practice of extra-curricular osteogenic sports.

It is well known that bone mass is around 60-80% genetically determined [2], therefore it is of importance to know which factors contribute to the development of bone mass. In this regard, weight bearing and high-impact intense PA, mainly through participation in sports, have shown osteogenic effects [4]. It has been shown that boys doing ≥ 3 h/week of extra-curricular sport increase total lean and bone mass to a greater extent than those not doing extra-curricular sports [5], which has been observed also in young [27] and adolescent girls [28]. In contrast, physical inactivity has been proposed as a determinant factor for low bone mass in young women [29].

Little is known about the association among sedentary behaviours and bone mass in a key period such as adolescence. In adolescents from the AVENA study, Vicente-Rodríguez et al. concluded that ≥3 h/day watching TV was associated with reduced total bone mass in boys, and their results suggested that extra-curricular PA could counteract this deleterious effect [14]. The present study do so taking into account a large list of sedentary behaviours (i.e. time spent on TV viewing, computer games, console games, internet for non-study, internet for study and study), additional DXA measurements in regions of clinical relevance regarding osteoporosis (i.e. lumbar spine and femoral neck) and controlling for relevant confounders that have been shown to be associated with bone mass during adolescence, such as lean mass [15] and objectively measured PA [3].

Our results showed that among the sedentary behaviours studied, total sedentary time in boys and time spent studying in girls were negatively associated with whole body BMC, respectively. However, these associations disappeared after controlling for lean mass, which has been strongly and positively associated with bone mass in this sample of adolescents [15]. Interestingly, the use of internet for non-study in boys and the time spent studying in girls were negatively associated with whole body and femoral neck BMC, respectively, even after controlling for lean mass and MVPA. In addition, it is important to notice that there was a negative borderline association between the total sedentary time and femoral neck BMC after controlling for lean mass and MVPA (models 2 and 3), supporting the main findings of this study.

The femoral neck, one of the regions with clinical relevance regarding osteoporosis, was significant and negatively associated with time spent studying in all models of adjustment in girls. Therefore, complementary analyses were performed at this site. The prevalence of girls with low femoral neck BMC was calculated considering both the time spent studying and the participation in extra-curricular sports. Our results showed that the prevalence of girls with low femoral neck BMC was smaller in those girls doing ≥3 h/week of extra-curricular osteogenic sports than in the rest of the sample (girls doing < 3 h/week of extra-curricular sports + girls doing non-osteogenic sports + girls not doing extra-curricular sport). Moreover, the cut-off for time of study (low: <3 h/day and high: ≥ 3 h/day) was also included in our analyses as in previous studies [14]. Similar results were obtained independently of the chosen cut-off. The analyses of other regions, such as whole body and lumbar spine showed also similar outcomes. These results suggest the importance of extra-curricular and osteogenic sports in the development of healthy bones, especially when sedentary behaviours, such as to stay seated for studying are evident. Similarly, it has been shown that sedentary occupations in adulthood are associated with an increased risk of hip fracture in elderly people [30].

The mechanisms by which sedentary behaviours lead to poor bone health are not well understood. According to a recent literature review [13], sedentary behaviour leads to a rapid increase in bone resorption without concomitant changes in bone formation, resulting in reduced BMC. The present results indicate that some sedentary behaviours can be more detrimental for bone health than others. Both the amount and the pattern of sedentary time may have influence on bone metabolism. In a recent study, we observed that the time studying was a good surrogate marker of objectively measured sedentary time (the higher the time studying the higher the time spent sedentary) in a large sample of European adolescents [19]. Moreover, sitting is common when a subject is studying or surfing internet. Compared with other sedentary activities, these activities are characterized by spending a lot of minutes in the same position. As a result of this, an excessive time without mechanical loading could be detrimental for bone health. We encourage adolescents to practice extra-curricular osteogenic sports at least 3 h/week in order to break the sedentary time caused by some sedentary behaviours such as study.

### Limitations and strengths

Although we controlled for several potential confounders we cannot be certain that other unmeasured confounders have not influenced our observations. Our study focus on adolescents from Zaragoza, Spain, since bone mass by DXA was only assessed in this sub-sample of the HELENA-CSS, so the conclusions cannot be generalized to whatever population. Cross-sectional studies only can provide suggestive evidence concerning causal relationships. However, in this specific case, it seems reasonable to think that time spent on sedentary behaviours could influence BMC, whereas it is not so clear the mechanisms by which bone mass could determine the time spent on sedentary behaviours.

The use of sophisticated methods, such as DXA to assess body composition, and the use of accelerometers to assess PA are strengths of the study. In addition, this study includes a rather complete set of confounders, i.e. height, sexual maturation, lean mass and MVPA, which is crucial to examine the current research question.

## Conclusion

The results of this report indicate that some sedentary behaviour, such as the use of internet for non-study (in boys) and the time spent studying (in girls) are negatively associated with whole body and femoral neck BMC, respectively. In addition, at least 3 h/week of extra-curricular osteogenic sports may help to counteract the negative association of time spent studying on bone health in girls. Additional studies (with a longitudinal or intervention design) must determine with more accuracy the public health importance of these findings.

## Competing interests

The authors declare that they have no competing interests.

## Authors’ contributions

All the authors have substantially contributed to this work: LGM, JPRL, LAM and GVR designed research; LGM, JPRL, LAM and GVR conducted research; LGM, JPRL and GVR performed statistical analysis; LGM and JPRL wrote paper; LGM, JPRL, LAM and GVR had primary responsibility for final content. All authors have read and approved the final manuscript.

## Pre-publication history

The pre-publication history for this paper can be accessed here:

http://www.biomedcentral.com/1471-2458/12/971/prepub
